# Characterization of the Temporal Pattern of Blood Protein Digestion in *Rhodnius prolixus*: First Description of Early and Late Gut Cathepsins

**DOI:** 10.3389/fphys.2020.509310

**Published:** 2021-01-13

**Authors:** Bianca Santos Henriques, Bruno Gomes, Pedro Lagerblad Oliveira, Elói de Souza Garcia, Patrícia Azambuja, Fernando Ariel Genta

**Affiliations:** ^1^Laboratory of Insect Physiology and Biochemistry, Oswaldo Cruz Institute – Oswaldo Cruz Foundation (IOC-FIOCRUZ), Rio de Janeiro, Brazil; ^2^National Institute of Science and Technology for Molecular Entomology (INCT-EM), Cidade Universitária, Rio de Janeiro, Brazil; ^3^Federal University of Rio de Janeiro, Rio de Janeiro, Brazil

**Keywords:** *Rhodnius prolixus*, protease, protein digestion, cathepsin L, cathepsin D

## Abstract

*Rhodnius prolixus* is one important vector for the parasite *Trypanosoma cruzi* in Latin America, where Chagas disease is a significant health issue. Although *R. prolixus* is a model for investigations of vector–parasite interaction and transmission, not much has been done recently to further comprehend its protein digestion. In this work, gut proteolysis was characterized using new fluorogenic substrates, including optimum pH, inhibition profiles, and tissue and temporal expression patterns. Each protease possessed a particular tissue prevalence and activity cycle after feeding. Cathepsin L had a higher activity in the posterior midgut lumen, being characterized by a plateau of high activities during several days in the intermediate phase of digestion. Cathepsin D showed high activity levels in the tissue homogenates and in the luminal content of the posterior midgut, with a single peak 5 days after blood feeding. Aminopeptidases are highly associated with the midgut wall, where the highest activity is located. Assays with proteinaceous substrates as casein, hemoglobin, and serum albumin revealed different activity profiles, with some evidence of biphasic temporal proteolytic patterns. Cathepsin D genes are preferentially expressed in the anterior midgut, while cathepsin L genes are mainly located in the posterior portion of the midgut, with specific sets of genes being differently expressed in the initial, intermediate, or late phases of blood digestion.

Significance Statement

This is the first description in a non-dipteran hematophagous species of a sequential protease secretion system based on midgut cathepsins instead of the most common insect digestive serine proteases (trypsins and chymotrypsins). The midgut of *R. prolixus* (Hemiptera) shows a different temporal expression of proteases in the initial, intermediate, and late stages of blood digestion. In this respect, a different timing in protease secretion may be an example of adaptative convergence in blood-sucking vectors from different orders. Expanding the knowledge about gut physiology in triatomine vectors may contribute to the development of new control strategies, aiming the blocking of parasite transmission.

## Introduction

*Rhodnius prolixus* (order Hemiptera, family Reduviidae, subfamily Triatominae) is an important vector of *Trypanosoma cruzi*, the etiological agent of Chagas disease that affects six to seven million people worldwide, mostly in Latin America (Coura, 2015). Due to its easy adaptability to the laboratory environment and artificial feeding, *R. prolixus* is considered as a traditional model for the study of insect physiology and parasite–vector interactions since the 1930s (Garcia et al., 2007). *R. prolixus* is mainly a hematophagous insect capable of ingesting enormous amounts of blood at once (Garcia et al., 2007). As other hemipteran insects, along with cucujiform coleopterans, *R. prolixus* differs from the most common model of insect protein digestion regarding the class of major gut proteases. Insects generally rely on serine proteases (trypsin and chymotrypsin) for food protein hydrolysis, whereas hemipterans use lysosomal-like acidic proteases in their digestion. The use of lysosomal enzymes is thought to have risen after the hemipteran sap-sucking common ancestor lost its digestive serine proteases while adapting to sugar-rich food requiring little to no protein digestion (Terra and Ferreira, 2012). In this regard, these insects are supposed to have mobilized originally intracellular proteases to a secretory pathway in the evolutionary readaptation to other food sources, which required the regain of the ability to digest proteins (Terra and Ferreira, 2012).

The characterization of *R. prolixus* digestive enzymes revealed activities of cathepsin D-like aspartic peptidases (EC 3.4.23.5), cathepsin L-like cysteine peptidases (EC 3.4.22.15) as well as exopeptidases, such as aminopeptidases and carboxypeptidases A and B (Houseman and Downe, 1983b; Terra et al., 1988). The study of these digestive proteases practically halted by the late 1980s and, since then, little was done to introduce new approaches to this critical aspect of the digestive physiology of *R. prolixus*.

The release and annotation of the genome of *R. prolixus* (Mesquita et al., 2015) provide an important opportunity to study digestion in this species. Data mining for protease sequences in the *R. prolixus* genome allowed the identification of over 400 genes in different protease families (Henriques et al., 2017). Moreover, phylogenetic analyses revealed gene expansion through recent gene duplication events in three protease families, namely, A1 (cathepsin D), C2 (calpain), and M17 (leucine aminopeptidase), that may be connected to the adaptation of hematophagous feeding in triatomines. The protease family C1 (cathepsin L) was also shown to be expanded, with a high number of gene copies. However, most of the cathepsin L duplications occurred before the divergence of the triatomine family (Henriques et al., 2017).

In the present study, we screened and characterized the digestive proteolytic activities of *R. prolixus* against new synthetic/proteinaceous substrates. We also analyzed the pattern of hydrolysis levels after the ingestion of blood using fluorogenic substrates for cathepsin L, cathepsin D, and aminopeptidase and the proteinaceous substrates casein, hemoglobin, and serum albumin. Genes of two expanded families (A1 and C1) were assessed for their preferential tissue expression, along with the pattern of relative expression throughout the digestive periods, both in the anterior and in the posterior portions of the midgut. The results provide evidences for the involvement of different proteases in the initial, intermediate or late stages of blood digestion and further the knowledge on the digestive physiology of *R. prolixus.*

## Materials and Methods

### Insects and Sample Preparation

All experiments were performed with male *R. prolixus* reared in the Laboratory of Insect Biochemistry and Physiology (IOC/Fiocruz) and maintained at 28°C with 60–70% relative humidity. Newly emerged starving male adults were fed, approximately 2 weeks after the final molt, with defibrinated rabbit blood through the latex membranes of a temperature-regulating apparatus (Garcia et al., 1984). Blood was provided by the Center of Creation of Animals, respecting the guidelines established by the Ethics Committee on Animal Use (CEUA/Fiocruz). Only fully engorged insects were used in the determinations.

For protease activity assessment and protein determination, the insect digestive tract was dissected from fasting insects and, at 2, 5, 7, 9, 12, and 14 days after feeding (daf), divided into the anterior midgut (AMG), posterior midgut (PMG), and hindgut (HG) (Terra et al., 1988). Each biological replicate consisted of tissues dissected from two individuals homogenized together. The actual numbers of replicates (*n*) are given in the figure legends. The AMGs were disrupted in 500 μl of 0.15 M NaCl, and tissues were manually separated from the content of this digestive compartment. The tissues were rinsed in saline solution to remove all remaining undigested blood. For the other gut compartments, PMG and HG were disrupted in 250 μl of 0.15 M saline each and centrifuged at 4°C for 5 min at 5,000 × *g* to separate the content (supernatant) from tissue (pellet) fractions (Waniek et al., 2014). All tissue pools were homogenized in 250 μl of 0.15 M saline using a pellet pestle cordless motor (Z359971 Sigma). The final volume of homogenates obtained from unfed insects and insects at 2, 5, and 7 daf was measured for the adjustment of calculations of the concentration for each sample (Supplementary Table 1). Control of the accuracy of the micropipettes resulted in measurements of 247 ± 1 and 500 ± 0 μl. The only samples that showed differences to controls larger than 5% were those of AMG at 2 and 5 daf. For calculations of samples obtained after 7 daf, the control values were used (see Supplementary Table 1).

### Proteolytic Assays With Synthetic Substrates

The activity of freshly prepared *R. prolixus* gut samples (see section “Insects and Sample Preparation”) obtained at different daf was tested with the synthetic substrates (Sigma, United States): (1) Z-Phe-Arg-AMC (Ref. C9521; λex 380 nm, λem 460 nm), used as cathepsin L substrate, (2) 7-methoxycoumarin-4-acetyl-Gly-Lys-Pro-Ile-Leu-Phe-Phe-Arg-Leu-Lys(DNP)-D-Arg-amide (Ref. M0938; λex 328 nm, λem 393 nm), used as cathepsin D substrate, and (3) L-Leu-AMC (Ref. L2145; λex 380 nm, λem 440 nm), used as aminopeptidase substrate. For cathepsin L and aminopeptidase, the assays were carried out with 25 μl of sample in a total volume of 200 μl and 10 μM of substrate. For cathepsin D, 15 μl of sample was assayed in a total volume of 50 μl and 10 μM of substrate (Pimentel et al., 2017). All assays were carried out in a black, opaque 96-well microplate (Costar 3915, Corning), and fluorescence was measured every 30 s for 1 h at 30°C (SpectraMax^®^ Gemini XPS, Molecular Devices). One micro-unit of peptidase activity (μU) is the amount of enzyme that releases 1 pmol of methyl coumarin or methoxycoumarin per minute.

### Effect of pH Variation and Specific Inhibitors

The effect of pH variation on the proteolytic activity on every substrate tested was verified using glycine-HCl, pH 3.6, sodium acetate, pH 3.6 and 4.0, citrate-phosphate, pH 4.0, 5.0, and 6.0, MES, pH 6.0 and 7.0 (Ref. M3885, Sigma), EPPS pH 7.3, 8.0, and 8.7 (Ref. E9502, Sigma), and glycine-NaOH, pH 9.0. The effects of peptidase inhibitors were tested for all substrates at the pH in which the enzymes displayed maximal activity. For this, we used ethylenediaminetetraacetic acid (EDTA) at final concentrations of 1 mM, 1,10-phenanthroline at 1 mM, trans-epoxysuccinyl-L-leucylamido(4-guanidino)butane (E-64) and (2*S*,3*S*)-trans-epoxysuccinyl-L-leucylamido-3-methylbutane (E-64c) at 10 μM, pepstatin A at 1 μM, phenylmethanesulfonyl fluoride (PMSF) at 1 mM, leupeptin at 100 μM, amastatin at 10 μM, bestatin at 20 or 60 nM, DL-dithiothreitol (DTT) at 10 mM, and ethylene-bis(oxyethylenenitrilo)tetraacetic acid (EGTA) at 1 mM, unless otherwise specified. During the inhibition assays, the samples were incubated for 30 min at 30°C with the inhibitors before the addition of the substrates. Both assays, controls without inhibitor and reactions with inhibitor, were carried as described in section “Proteolytic Assays with Synthetic Substrates”.

### Determination of Concentrations of Proteins and Protease Activity Using Proteinaceous Substrates

The total concentration of proteins was assessed using Bio-Rad Protein Assay Dye Reagent Concentrate (Ref. #500-0006) at 595 nm.

Total proteolytic activities from fresh samples were estimated using bovine casein (Ref. C7078, Sigma), bovine serum albumin (Ref. A2153, Sigma), and bovine hemoglobin (Ref. H2500, Sigma). For each protein cited above, 220 μl of a solution of 2% (w/v) of the substrate in 0.1 M citrate-phosphate buffer, pH 5.5, was added to 220 μl of tissue sample and incubated for 4 h at 30°C. Aliquots of 80 μl were taken at 0, 1, 2, 3, and 4 h and transferred to a fresh tube, where we terminated the reaction by adding 80 μl of 10% (w/v) trichloroacetic acid solution. All terminated reaction samples were centrifuged at room temperature for 5 min at 10,000 × *g*, and the released free peptides were quantitated in the supernatant. Briefly, 10 μl of the supernatant was transferred to a clear, flat-bottomed, 96-well microplate (Ref. 353047, Falcon) and filled to 50 μl with H_2_O. Then, 125 μl of 0.5 M Na_2_CO_3_ was added to each well, followed by 25 μl of fivefold-diluted Folin and Ciocalteu’s phenol reagent (Ref. F9252, Sigma). The plates were incubated at 37°C for 30 min and read at 660 nm (SpectraMax 190^®^, Molecular Devices). One unit was determined as the amount of enzyme that hydrolyzed protein to produce color equivalent to 1 μmol of tyrosine (Anson, 1938).

### Gene Expression Analysis

For RT-PCR, salivary glands (SG), AMG, PMG, and HG were dissected and stored in RNA*later*^®^ Stabilization Solution (Ref. AM7024, ThermoFisher) at −80°C. Each pool consisted of tissue collected from six males and organized by digestion period: (i) unfed, (ii) early digestion: 2 and 5 daf, (iii) intermediate digestion: 7 and 9 daf, and (iv) late digestion: 12 and 14 daf. Then, mRNA was extracted using the ReliaPrep^TM^ RNA Tissue Miniprep System kit (Ref. Z6111, Promega), following the manufacturer’s instructions for non-fibrous tissues. Reverse transcription of cDNA was accomplished with SuperScript^TM^ III First-Strand Synthesis System for RT-PCR (Ref. 18080-051, Invitrogen), following the kit protocol.

The PCR reaction included 5 ng template cDNA, 0.2 mM dNTP mix (Ref. U1330, Promega), 0.5 μM each primer (forward and reverse), 4 μl 5 × Green GoTaq^®^ reaction buffer (Ref. M891A, Promega), 1.5 mM MgCl_2_ (Ref. A351H, Promega), 0.1 μl GoTaq^®^ DNA polymerase 5 U/μl (Ref. M829B, Promega), and filled with water up to 20 μl (Ref. W4502, Sigma). The primers were previously described (Henriques et al., 2017).

The PCR conditions were as follows: initial denaturation at 95°C for 2 min, followed by cycles of 95°C for 30 s, 55°C for 30 s, and 72°C for 1 min and a final extension step of 72°C for 5 min. The samples were resolved on 1% (w/v) agarose in tris/acetic acid/EDTA (Ref. #1610743, Bio-Rad) stained with 1 μg/ml ethidium bromide and viewed with an ultra-violet light transilluminator (E-Gel Imager, Life Technologies). A 100-bp DNA ladder (Ref. 76712, Affymetrix) was run on every gel to confirm the expected size of the amplification product. Gel band intensity was estimated semi-quantitatively using ImageJ software version 1.46r, and relative gene expressions were calculated as a ratio of the band intensities of the target genes to that of the constitutive gene, *R. prolixus* ribosomal 18S (GenBank ID: AJ421962).

### Statistical Analysis

All statistical analyses were conducted using the statistic software GraphPad Prism v6.04 (CA, United States) for Microsoft Windows, using one or two-way ANOVA tools. The significance levels were considered as statistically significant when *p* < 0.05.

## Results

### Activity Characterization With Fluorogenic Substrates

**FIGURE 1:**
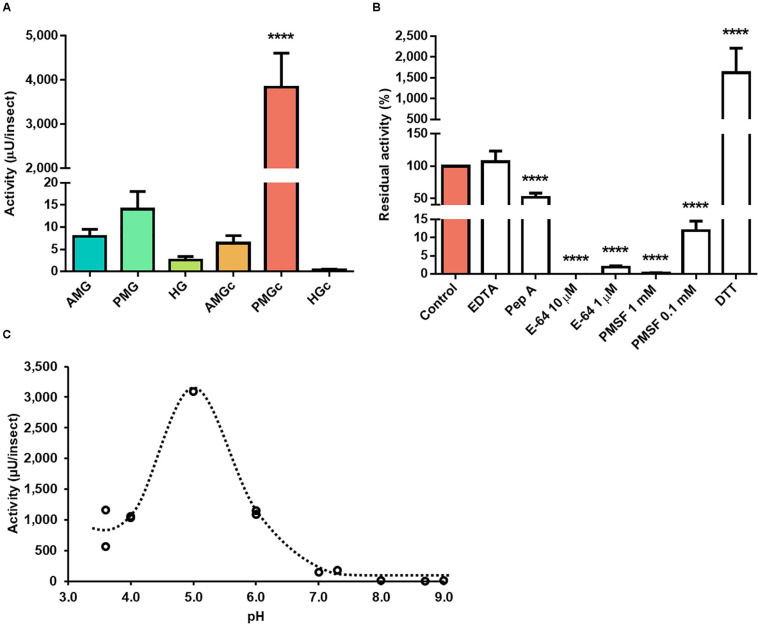
Compartmentalization and characterization of cathepsin L activity in the digestive tract compartments of male adult *Rhodnius prolixus*. **(A)** Comparison of protease activity between homogenates of the anterior midgut (AMG), posterior midgut (PMG), and hindgut (HG) and their respective luminal contents, assayed in 0.2 M citrate-phosphate pH 5.5. **(B)** Effects of the protease inhibitors EDTA (1 mM), pepstatin A (1 μM), E-64 (10 and 1 μM), PMSF (1 mM and 0.1 mM), and the activator DTT (10 mM) on the proteolytic activity of the PMG contents, assayed in 0.2 M citrate-phosphate pH 5.5. **(C)** Effects of the pH variation on the proteolytic activity of the posterior midgut contents using the buffers glycine-HCl, pH 3.6; sodium acetate, pH 3.6 and 4.0; citrate-phosphate, pH 4.0, 5.0, and 6.0; MES, pH 6.0 and 7.0; EPPS, pH 7.3, 8.0, and 8.7; and glycine-NaOH, pH 9.0. Bars and markers are means of at least three biological replicates with the standard error of the mean. Asterisks denote statistically significant differences, where ****p ≤ 0.0001.

The proteolytic activity on cathepsin L substrate was highest in the luminal content of the posterior midgut of adult male *R. prolixus* (Figure 1A). In this portion, the activity on this substrate was at least 200-fold higher than in the tissue homogenates and other luminal content preparations. While EDTA had no significant effect on the PMG content activity, pepstatin A caused a reduction of about 48% (Figure 1B). Both E-64 and PMSF abolished the protease activity in the highest recommended usage concentrations of 10 μM and 1 mM, respectively (Figure 1B). However, in the presence of 1 μM E-64, residual activity increased from 0.03% to about 2%, whereas the residual activity in the presence of 0.1 mM PMSF increased from 0.24% to about 12% (Figure 1B). As expected for a cysteine protease, the addition of DTT induced a 15-fold increase in hydrolytic activity over Z-F-R-AMC, and pH variation indicated a maximal proteolytic activity at pH 5 (Figure 1C).

**FIGURE 2:**
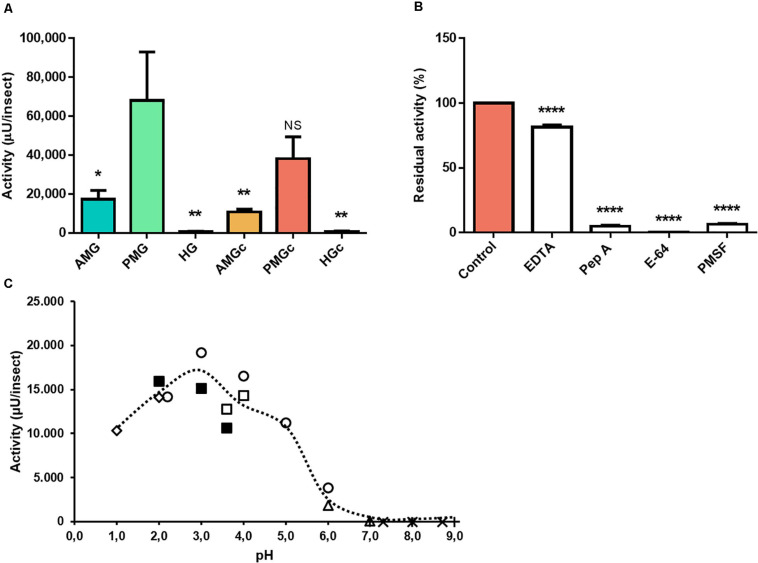
Compartmentalization and characterization of cathepsin D activity in the digestive tract compartments of male adult *Rhodnius prolixus*. **(A)** Comparison of protease activity between homogenates of the anterior midgut (AMG), posterior midgut (PMG), and hindgut (HG) tissues and their respective luminal contents, assayed in 0.2 M glycine-HCl, pH 3.6. **(B)** Effects of the protease inhibitors EDTA (1 mM), pepstatin A (1 μM), E-64 (10 μM), and PMSF (1 mM) on the proteolytic activity of PMG contents, assayed in 0.2 M glycine-HCl, pH 3.6. **(C)** Effects of the pH variation on the proteolytic activity of PMG content samples, using the buffers KCl, pH 1.0 and 2.0 (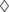); glycine-HCl, pH 2.0, 3.0, and 3.6 (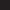); sodium acetate, pH 3.6 and 4.0 (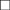); citrate-phosphate, pH 2.2, 3.0, 4.0, 5.0, and 6.0 (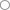); MES pH 6.0 and 7.0 (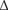); EPPS pH 7.3, 8.0, and 8.7 (×); and Tris-HCl, pH 7.0, 8.0, and 9.0 (+). Bars and markers are means of at least three biological replicates with the standard error of the mean (SEM). Asterisks denote statistically significant differences. NS, *p* > 0.05; *p ≤ 0.05; **p ≤ 0.01; ****p ≤ 0.0001.

The use of the cathepsin D substrate resulted in the highest levels of enzyme activity recorded herein. The major activities occurred in the tissue homogenate of the PMG, followed by the PMG contents (Figure 2A). In the presence of different protease class inhibitors, the activity of PMG tissue on this substrate demonstrated an unusual behavior. EDTA did not influence the activity levels, and pepstatin A and E-64 led to total inhibition (*n* = 3, *p* ≤ 0.01). PMSF resulted in residual activity of 36 ± 16% (Figure 2B). Maximal activity occurred at pH 3.6 (Figure 2C).

**FIGURE 3:**
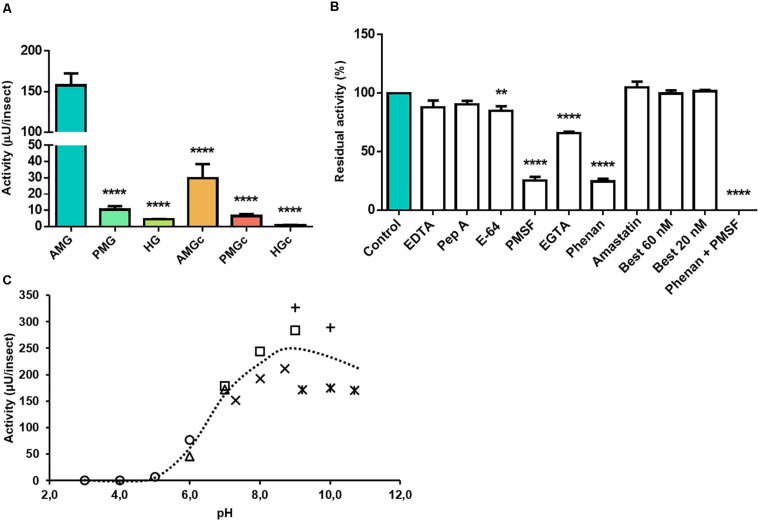
Compartmentalization and characterization of aminopeptidase activity in the digestive tract compartments of male adult *Rhodnius prolixus*. **(A)** Comparison of protease activity between homogenates of the anterior midgut (AMG), posterior midgut (PMG), and hindgut (HG) tissues and their respective luminal contents, assayed in 0.2 M glycine-NaOH pH 9.0. **(B)** Effects of the protease inhibitors EDTA (10 mM), pepstatin A (1 μM), E-64 (10 μM), PMSF (1 mM), EGTA (10 mM), phenanthroline (10 mM), amastatin (10 μM), bestatin (20 and 60 nM), and a combination of phenanthroline and PMSF, in their respective concentrations, on the proteolytic activity of AMG tissues, assayed in 0.2 M glycine-NaOH pH 9.0. **(C)** Effects of pH variation on the proteolytic activity of AMG tissue homogenate, using the buffers citrate-phosphate pH 3.0, 4.0, 5.0, and 6.0 (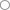); MES, pH 6.0 and 7.0 (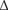); EPPS, pH 7.3, 8.0, and 8.7 (×); Tris-HCl, pH 7.0, 8.0, and 9.0 (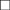); glycine-NaOH, pH 9.0 and 10.0 (+); and carbonate/bicarbonate 9.2, 10.0, and 10.7 (*). Bars and markers are means of at least nine biological replicates with the standard error of the mean (SEM). Asterisks denote statistically significant differences, **p ≤ 0.01 and ****p ≤ 0.0001.

The highest activity against aminopeptidase substrate was located in the AMG tissue (*n* = 3, *p* ≤ 0.0001) (Figure 3A). EDTA generated no significant effect on substrate hydrolysis, but EGTA and phenanthroline resulted in reductions in protease activity, leading to 66 ± 1 and 25 ± 3% residual activities, respectively (Figure 3B). E-64 reduced proteolysis by about 15%, and PMSF drastically inhibited 75% of this activity. The presence of both phenanthroline and PMSF led to complete inhibition (Figure 3B). Bestatin (inhibitor of leucine aminopeptidase and aminopeptidase B) did not affect L-Leu-AMC hydrolysis in the AMG tissue samples. Amastatin (inhibitor of leucine aminopeptidase and aminopeptidase A) led to no significant change in activity (Figure 3B). Activity was maximal in a neutral to alkaline broad pH range, with the highest hydrolysis levels at pH 9 (Figure 3C).

### Proteolytic Activity Patterns During Blood Digestion in *R. prolixus*

For characterizing the hydrolysis process of proteins during blood digestion, we followed the activity against the three fluorogenic substrates mentioned above by analysis of the digestive tract compartments of male insects, including their respective luminal contents, in the course of 14 days.

At 14 daf, only a small amount of blood still remained in the anterior midgut (Supplementary Figure 1), and the concentration of proteins returned to levels close to the unfed-state levels, thus characterizing the final stage of digestion (Supplementary Figure 2 and Figure 2D). Both in the AMG and PMG tissue homogenates, the concentration of proteins increased at 2 daf and was maintained at steady levels throughout the whole digestion process (Supplementary Figures 2A,B). In the HG tissues, however, the total concentration of proteins oscillated during the digestive process, with a statistically significant decrease at 2 daf (Supplementary Figure 2C). As expected, the concentration of proteins of the luminal fraction of the AMG displayed an almost 750-fold increase due to the blood, which slowly diminished as the blood was digested (Supplementary Figure 2D). In the PMG contents, the concentration of proteins peaked at 2 daf (*n* = 21, *p* ≤ 0.0001) and then steadily decreased on the following days until reaching basal levels at 14 days (Supplementary Figure 2E). In the luminal content of the HG, the protein levels remained low and statistically similar to each other throughout the whole process (Supplementary Figure 2F). Furthermore, the protein concentrations in the AMG contents decreased at a constant rate from day 2 to day 12 (Supplementary Figure 3A), with a significant decrease in the rate of protein digestion at day 14, reinforcing the end of this physiological cycle (Supplementary Figure 3B). The total protein amounts of the contents of the anterior midgut are shown in Supplementary Table 3.

**FIGURE 4:**
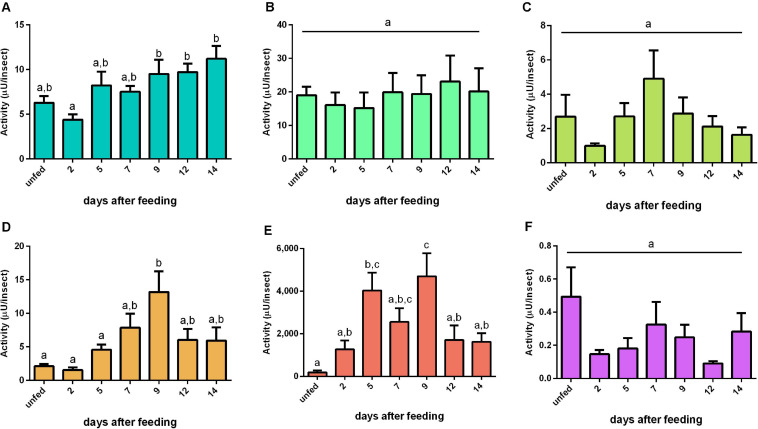
Cathepsin L-like activity (μU per insect), using Z-Phe-Arg-AMC as substrate, in the different digestive compartments (tissues and luminal contents) of *Rhodnius prolixus* male adults, unfed and at 2, 5, 7, 9, 12, and 14 daf on defibrinated rabbit blood, assayed in 0.2 M citrate-phosphate buffer, pH 5.5. **(A)** Anterior midgut tissues. **(B)** Posterior midgut tissues. **(C)** Hindgut tissues. **(D)** Anterior midgut contents. **(E)** Posterior midgut contents. **(F)** Hindgut contents. Figures are means ± SEM (*n* = 9). In a dataset, groups with the same superscript letter are not significantly different (*p* > 0.05). Statistical analysis consisted of ordinary one-way ANOVA with Tukey’s multiple-comparisons test. Consider different scalings of activities.

The activity of cathepsin L-like had no significant changes during the digestion period in PMG and HG tissues or HG contents (Figures 4B,C,F). In the AMG tissues, the activity slightly decreased at 2 daf and then gradually increased, culminating in statistically different levels of proteolysis from 9 until 14 daf (*n* = 9, *p* ≤ 0.05) (Figure 4A). In the AMG contents, the activity reached a peak at 9 daf (Figure 4D). The major activity occurred in the luminal content of the PMG contents (Figure 4E). The proteolysis in this sample reached the highest level of activity in the interval between 5 and 9 daf, with a statistically not significant low value at 7 daf (Figure 4E).

The cathepsin L-like-specific activities in AMG tissues and PMG contents followed a pattern similar to their respective total activity determinations per insect (Supplementary Figures 4A,E). In contrast, PMG tissues and HG contents had higher specific activities in unfed insects (Supplementary Figures 4B,F), and HG tissue homogenates only between 12 and 14 daf (Supplementary Figure 4C). In the AMG contents, the high concentrations of protein from the blood caused a distortion in the pattern of specific activity, with a substantial drop at 2 daf (*p* ≤ 0.0001) and no statistical difference among the other time frames (Supplementary Figure 4D).

**FIGURE 5:**
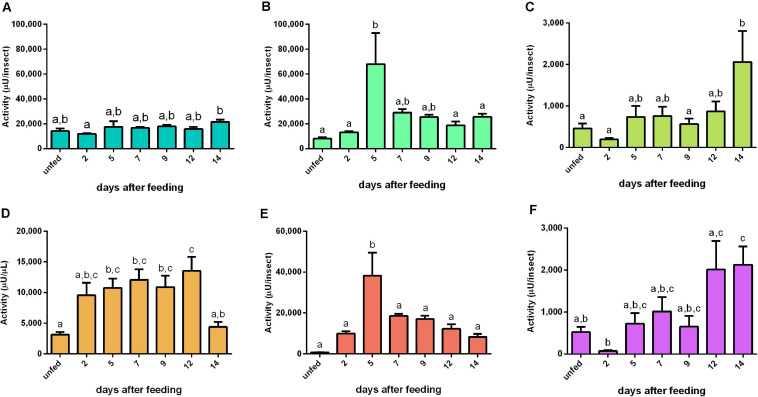
Cathepsin D-like activity (μU per insect) in the different digestive compartment tissues and luminal contents of *Rhodnius prolixus* male adults, unfed and at 2, 5, 7, 9, 12, and 14 daf on defibrinated rabbit blood, assayed in 0.2 M glycine-HCl buffer, pH 3.6. **(A)** Anterior midgut tissues. **(B)** Posterior midgut tissues. **(C)** Hindgut tissues. **(D)** Anterior midgut contents. **(E)** Posterior midgut contents. **(F)** Hindgut contents. Figures are means ± SEM from nine different homogenate samples obtained from pools of two insects each. In a dataset, groups with the same superscript letter are not significantly different (*p* > 0.05). Statistical analysis consisted of ordinary one-way ANOVA with Tukey’s multiple-comparisons test. Consider different scalings of activities.

For the cathepsin D substrate, the activity in the AMG epithelium had a similar pattern with the cathepsin L-like activity in the same tissue (Figures 4A, 5A). In the AMG contents, this proteolytic activity had elevated levels from 2 to 12 daf, after which the cathepsin D-like activity returned to the basal levels found in unfed insects (Figure 5D). Both posterior midgut fractions depicted a significant peak of activity at 5 daf (Figures 5B,E). Furthermore, it is noteworthy that the tissue samples from the three regions of the midgut (AMG, PMG, and HG) showed proteolytic levels comparable to those observed in the respective secreted luminal contents. On the other hand, HG tissues and HG contents showed a low proteolytic activity against this substrate; however, both fractions displayed similar patterns, in which the activity reached the highest levels during late digestion (12–14 days) (Figures 5C,F). No significant changes in the specific activities of tissue homogenates were observed (Supplementary Figures 5A–C). The specific activity patterns of PMG and HG content samples were similar to Figures 5E,F (Supplementary Figures 5E,F), while for AMG contents the blood protein distorted the protease-specific activities (Supplementary Figure 5D).

**FIGURE 6:**
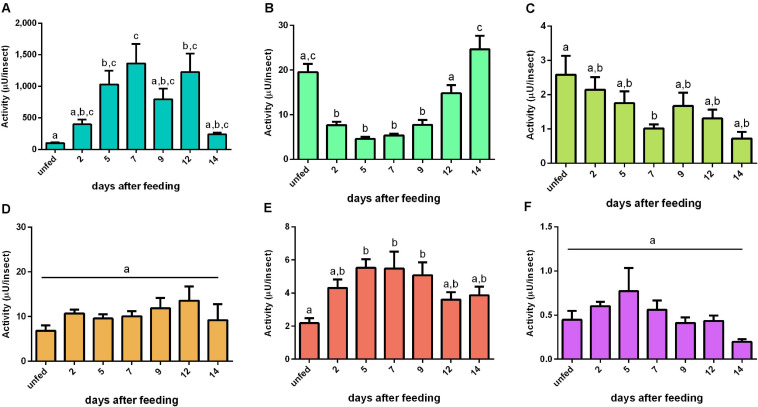
Aminopeptidase activities (μU per insect) in the different digestive compartment tissues and luminal contents of *Rhodnius prolixus* male adults, unfed and at 2, 5, 7, 9, 12, and 14 daf on defibrinated rabbit blood, assayed in 0.2 M glycine-NaOH buffer, pH 9. **(A)** Anterior midgut tissues. **(B)** Posterior midgut tissues. **(C)** Hindgut tissues. **(D)** Anterior midgut contents. **(E)** Posterior midgut contents. **(F)** Hindgut contents. Figures are means ± SEM from nine different homogenate samples obtained from pools of two insects each. In a dataset, groups with the same superscript letter are not significantly different (*p* > 0.05). Statistical analysis consisted of ordinary one-way ANOVA with Tukey’s multiple-comparisons test. Consider different scalings of activities.

Aminopeptidase activity was mainly located in the tissue homogenates of the AMG. In these samples, the activity increase was triggered by the blood intake, reaching a plateau at 5 daf and with minor oscillations until 12 daf (Figure 6A). No significant changes in the AMG contents were observed (Figure 6D). In the PMG tissue, activity was significantly reduced during the early and intermediate periods of digestion (2–5 and 7–9 days) and returned to unfed levels in 12–14 days (Figure 6B). The PMG contents had a pattern complementary to the tissue activity (Figure 6E). The aminopeptidase activities in the HG tissue had a descending pattern from unfed to 7 daf, whereas the activities of HG contents did not change (Figures 6C,F, respectively). Regarding specific activities, the AMG tissues (Supplementary Figure 6A) had similarities to the recorded activities per insect (Figure 6A). The specific activities of PMG tissue, AMG contents, and PMG contents were higher in unfed animals, with a drastic reduction after blood ingestion and a slow increase in the late digestive period (Supplementary Figures 6B,D,E). The HG tissues at 2 daf had activities higher than those from later samples but similar to the samples from unfed insects (Supplementary Figure 6C). No changes were observed in HG contents (Supplementary Figure 6F).

### Patterns of Activity Against Proteinaceous Substrates

With the purpose of understanding the relationships between activities against specific substrates and general proteolytic patterns, we assayed the digestive proteases of *R. prolixus* adults using casein, hemoglobin, and serum albumin. Hindgut was not included due to its low proteolytic activity.

**FIGURE 7:**
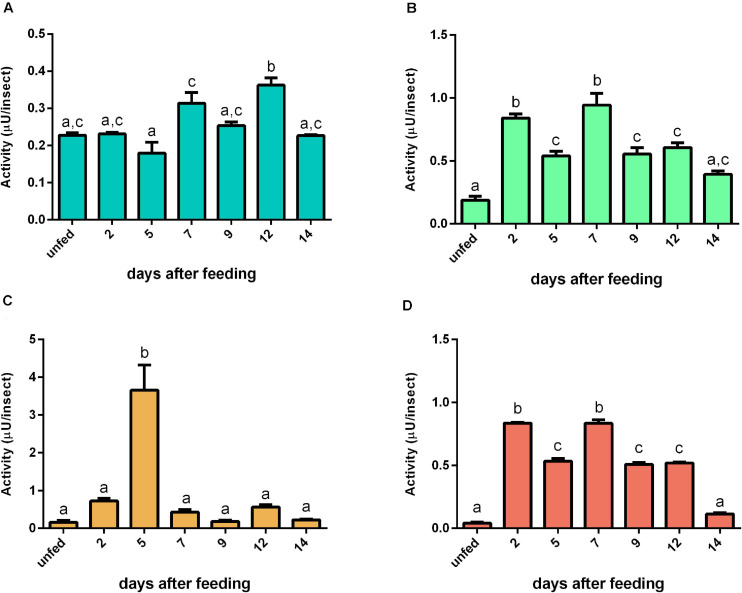
The proteolytic activity (μU per insect) on casein in the different digestive compartment tissues and luminal contents of *Rhodnius prolixus* male adults, unfed and at 2, 5, 7, 9, 12, and 14 daf on defibrinated rabbit blood, assayed in 0.2 M citrate-phosphate buffer, pH 5.5. **(A)** Anterior midgut tissues. **(B)** Posterior midgut tissues. **(C)** Anterior midgut contents. **(D)** Posterior midgut contents. Figures are means ± SEM from three different homogenate samples obtained from pools of two insects each. In a dataset, groups with the same superscript letter are not significantly different (*p* > 0.05). Statistical analysis consisted of ordinary one-way ANOVA with Tukey’s multiple-comparisons test. Consider different scalings of activities.

For bovine casein, the proteolytic activity in AMG tissue peaked at 12 daf (Figure 7A). In AMG contents, the proteolytic activity at 5 daf was higher than the activity from other days (Figure 7C). Both PMG fractions displayed similar proteolysis levels characterized by two proteolytic activity peaks at 2 and 7 daf (Figures 7B,D, respectively). Regarding specific activities, there were no significant changes in most values of AMG tissue samples, with only samples at 5 and 12 daf differing from each other significantly (Supplementary Figure 7A). In AMG contents, the samples after feeding showed lower specific activities than the samples from unfed insects (Supplementary Figure 7C). In PMG tissues, the activity at 7 daf was higher than the activities of unfed insects and at 5 daf (Supplementary Figure 7C). In PMG contents, there were no significant differences between the samples (Supplementary Figure 7D).

**FIGURE 8:**
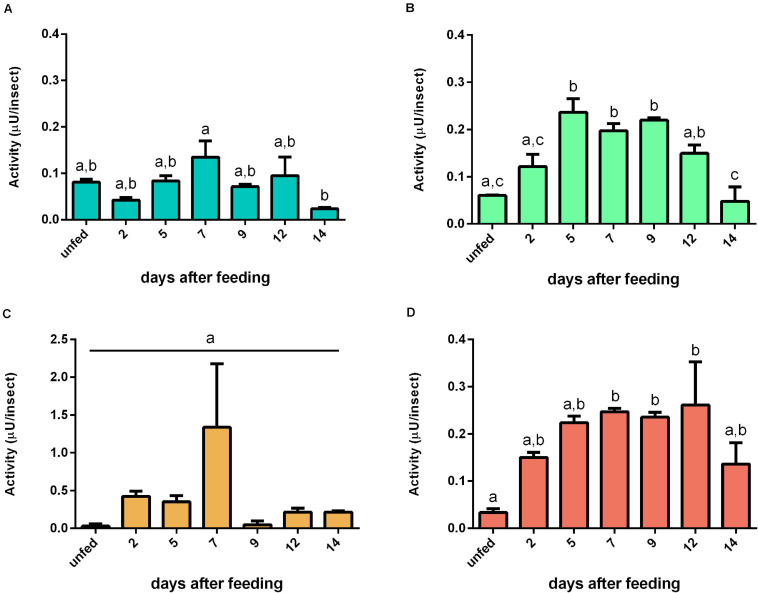
The proteolytic pattern of activity per insect against bovine hemoglobin in the different digestive compartment tissues and luminal contents of *Rhodnius prolixus* male adults, unfed and at 2, 5, 7, 9, 12, and 14 daf on defibrinated rabbit blood, assayed in 0.2 M citrate-phosphate buffer, pH 5.5. **(A)** Anterior midgut tissues. **(B)** Posterior midgut tissues. **(C)** Anterior midgut contents. **(D)** Posterior midgut contents. Figures are means ± SEM from three different homogenate samples obtained from pools of two insects each. In a dataset, groups with the same superscript letter are not significantly different (*p* > 0.05). Statistical analysis consisted of ordinary one-way ANOVA with Tukey’s multiple-comparisons test. Consider different scalings of activities.

In comparison to casein, we observed a different proteolytic pattern using bovine hemoglobin as substrate. The AMG tissue had only a significant decrease at 14 daf (Figure 8A). The PMG tissue had a higher activity plateau from 5 until 9 daf, with activity returning to the unfed levels at 12 daf (Figure 8B). The PMG contents had a similar pattern, with a higher activity plateau from 7 until 12 daf (Figure 8D). The AMG contents had no significant changes in activity along with time (Figure 8C). Regarding specific activities against hemoglobin, most values from AMG tissues were not significantly different from each other, with only the value at 7 daf being significantly different from those at 2 and 14 daf (Supplementary Figure 8A). The same observation was made in activities from PMG tissues, with only the value at 9 daf being significantly different from values at 2 and 14 daf. The results for AMG and PMG contents did not change significantly along with time (Supplementary Figures 8C,D).

**FIGURE 9:**
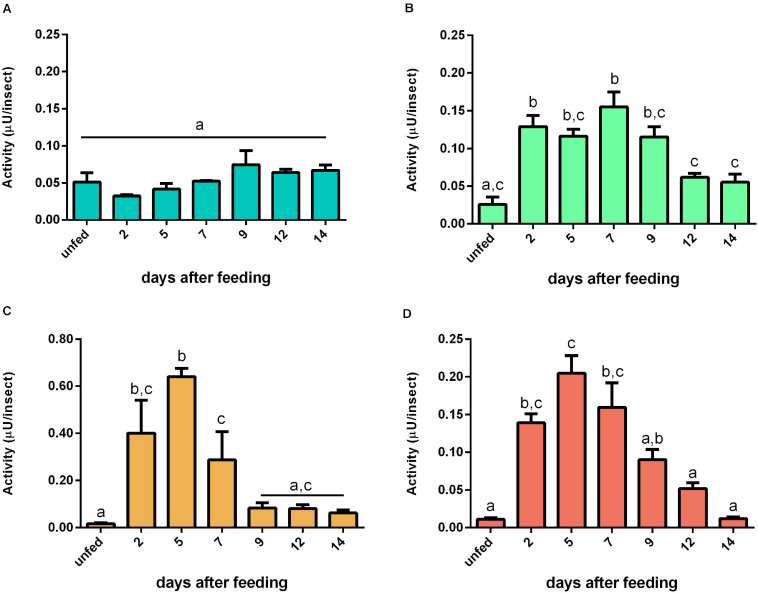
Activity (μU per insect) against bovine serum albumin in the different digestive compartment tissues and luminal contents of *Rhodnius prolixus* male adults, unfed and at 2, 5, 7, 9, 12, and 14 daf on defibrinated rabbit blood, assayed in 0.2 M citrate-phosphate buffer, pH 5.5. **(A)** Anterior midgut tissues. **(B)** Posterior midgut tissues. **(C)** Anterior midgut contents. **(D)** Posterior midgut contents. Figures are means ± SEM from three different homogenate samples obtained from pools of two insects each. In a dataset, groups with the same superscript letter are not significantly different (*p* > 0.05). Statistical analysis consisted of ordinary one-way ANOVA with Tukey’s multiple-comparisons test.

Regarding activity against bovine serum albumin, no significant changes were observed in the AMG tissues (Figure 9A). In contrast, the proteolytic levels had an abrupt increase at 2 daf in the other samples and returned to unfed values at 9 daf (Figures 9B–D). For the specific activities, no significant changes were seen in AMG and PMG tissues and PMG contents (Supplementary Figures 9A,B,D). In AMG contents, the results were affected by protein from the blood (Supplementary Figure 9C).

### Gene Expression Analyses

Gene expression analysis was carried out to infer the potential role of proteases from families A1 and C1 in the activity patterns described above. A previous analysis of the genome of *Rhodnius prolixus* revealed the presence of 18 and 16 genes of family A1 and C1, respectively (Mesquita et al., 2015; Henriques et al., 2017). These protease gene families were especially relevant here because the aspartic protease family A1 includes cathepsin D and cathepsin D-like proteins, and the cysteine protease family C1 includes cathepsin L and cathepsin L-like proteins (Rawlings et al., 2018). For brevity, *R. prolixus* genes in the cathepsin D family (A1) were numbered from A1 to A18 and in the cathepsin L family (C1) from C1 to C16. Supplementary Table 2 shows a list of their identification in the *R. prolixus* annotated genome (Vector Base IDs).

Genes A2, A3, A4, A5, A6, A10, A11, A14, A15, A17, and A18 exhibited a higher level of expression in AMG than in the other tissues, corresponding to 61% of the genes in this family (Table 1). Genes A9 and A16 showed a higher expression in AMG and HG; A7, A12, and A13 did not present a clear tissue preference, whereas only A1 was preferentially expressed in the PMG. Interestingly, gene A8 had splice variants with a different expression throughout the digestive tissues. The A8 shorter variant was more expressed in both midgut compartments, while the longer variant was more expressed in SG and HG (Supplementary Figure 10, left column, and Table 1).

**TABLE 1:**
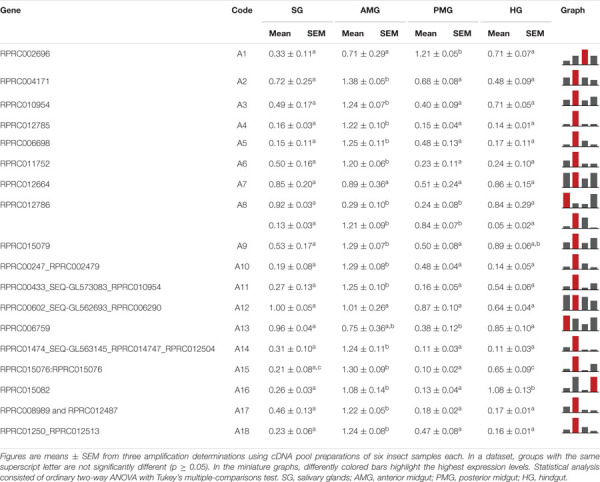
Relative expression patterns of genes from protease family A1 in the tissues of *Rhodnius prolixus* adult males.

In the AMG, A2, A3, A5, A8, A16, A17, and A18 did not show any differences in expression among the digestion phases (Table 2). A1, A4, A12, and A13 showed higher expressions in early digestion (Table 2). A6, A10, A11, A14, and A15 were primarily expressed in unfed insects, with a reduction after feeding, and gradually returned to unfed levels in the following days. A7 had a similar pattern, yet its relative expression was higher in the late phase (Table 2).

**TABLE 2:**
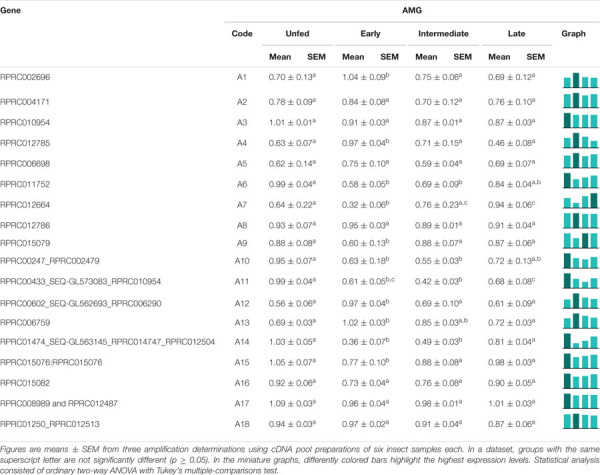
Relative expression of genes from protease family A1 in the anterior midgut of *Rhodnius prolixus* adult males, unfed and after feeding on defibrinated rabbit blood, including early (2–5 daf), intermediate (7–9 daf), and late (12–14 daf) digestion.

In the PMG, the expression of 44% of family A1 genes (A1, A3, A6, A9, A14, A15, A16, and A17) did not vary throughout digestion (Table 3). A5, A11, A12, and A13 were mainly expressed during early digestion, and A8 and A18 also had increased relative expression in the early stage but were not reduced in the subsequent phases (Table 3). Genes A4 and A7 had higher relative expression levels in the late stages of blood digestion, and A10 revealed a higher expression in unfed insects. Gene A2 presented a higher expression during *R. prolixus* unfed state and early digestion (Table 3).

**TABLE 3:**
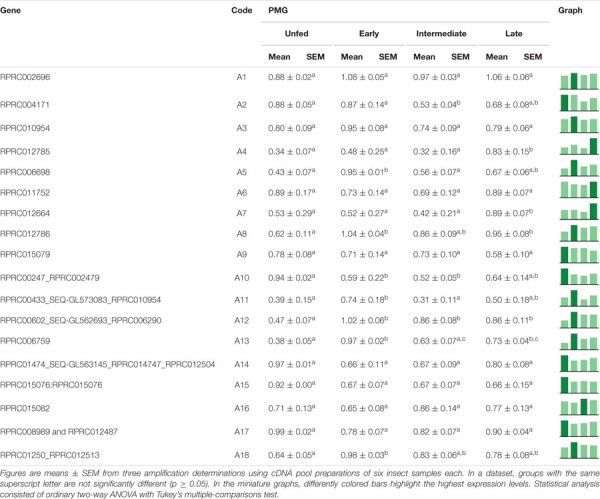
Relative expression of genes from protease family A1 in the posterior midgut of *Rhodnius prolixus* adult males, unfed and after feeding on defibrinated rabbit blood, including early (2–5 daf), intermediate (7–9 daf), and late (12–14 daf) digestion.

Among the family of C1 genes, C7, C11, C12, C13, C14, and C16, which correspond to 37% of the total, did not present significant differences in expression when comparing the different tissues (Table 4). C1, C3, C4, and C10 were expressed mostly, or even exclusively, in the posterior midgut. C6 was preferentially expressed in the hindgut, while C9 had a significantly higher expression in the AMG. C8 was transcribed mainly in the AMG and PMG (Table 4). Gene C5 presented evidence for alternative splicing, in which the larger amplicon was only found in the SG and HG, whereas the shorter product showed a significantly higher expression restricted in PMG (Supplementary Figure 11 and Table 4).

**TABLE 4:**
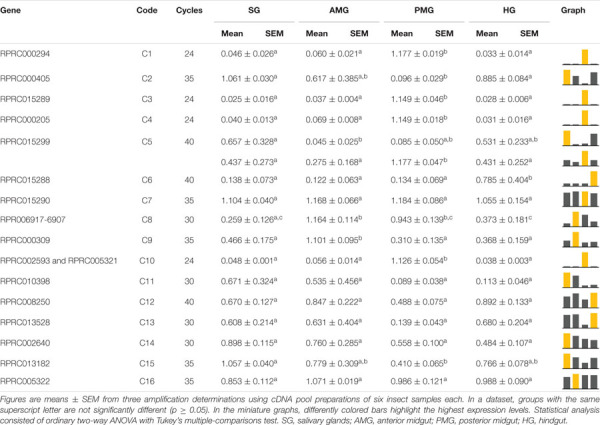
Relative expression patterns of genes from protease family C1 in the tissues of *Rhodnius prolixus* adult males.

In the AMG, only C2, C13, and C14 expression suffered no significant changes during the experimental period (Table 5). C5, C6, C7, and C16 were preferentially expressed in unfed insects. C15 was highly expressed in unfed insects, with a slight increase in early digestion and a gradual decrease and a significant reduction in the late period (Table 5). C1, C4, C8, C10, C11, and C12 expression declined in the intermediate stage (7–9 days), after which it returned to previous levels in late digestion (Table 5). Genes C3 and C9 presented different splicing in the AMG. While the former had one variant mainly expressed in unfed insects and the other during early and intermediate digestion stages, in the latter, both variants showed higher relative expressions previous to blood intake, which reduced during early and intermediate digestive phases and returned to unfed levels in late digestion (Supplementary Figure 11 and Table 5).

**TABLE 5:**
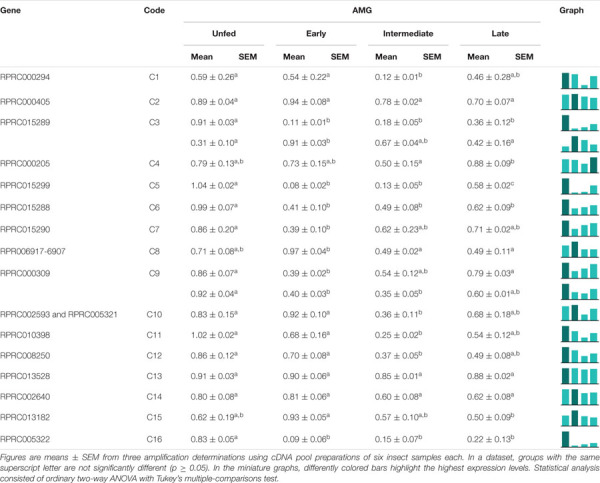
Relative expression of genes from protease family C1 in the anterior midgut of *Rhodnius prolixus* adult males, unfed and after feeding on defibrinated rabbit blood, including early (2–5 daf), intermediate (7–9 daf), and late (12–14 daf) digestion.

In the PMG, genes C2, C3, C4, C6, C8, C10, C11, C12, and C13, constituting 50% of the total, were equally expressed along the time slots (Table 6). Genes C1, C5, C7, C9, and C14 had reduced expression levels during the intermediate phase, with higher levels in late digestion (Table 6). Only gene C15 depicted a significantly higher expression in early digestion, and gene C16 gradually increased its relative expression, with a peak in the late digestion phase (Table 6).

**TABLE 6:**
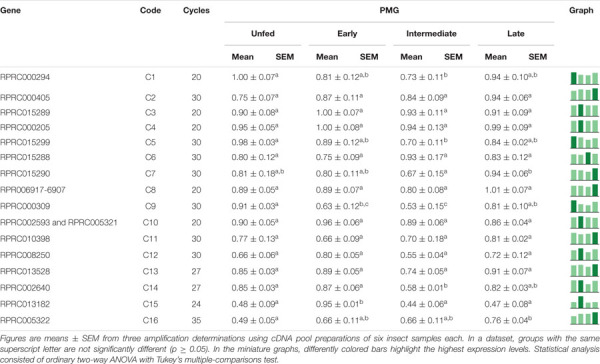
Relative expression of genes from protease family C1 in the posterior midgut of *Rhodnius prolixus* adult males, unfed and after feeding on defibrinated rabbit blood, including early (2–5 daf), intermediate (7–9 daf), and late (12–14 daf) digestion.

## Discussion

To our knowledge, this is the first time that different pools of proteases involved in initial, intermediate, or late stages of blood digestion were described outside the order Diptera. *R. prolixus* is a classic model in insect physiology, and studies on its proteolytic digestive system started in the late 1960s (Gooding, 1968, 1969). Some studies focused on *R. prolixus* midgut protease secretion (Persaud and Davey, 1971; Garcia and Garcia, 1977) and characterization of cathepsin L and cathepsin D-like activities (Terra et al., 1988). Recent genomic studies increased the knowledge about *R. prolixus* proteases, pointing to the relevance of both cathepsins in blood digestion (Henriques et al., 2017). However, most characterizations were achieved exclusively through colorimetric techniques, which are usually less specific or sensitive. Therefore, we started our analyses with a screening of the proteolytic activities in the different regions plus tissues and contents of the digestive tract of *R. prolixus* using fluorogenic substrates. It is important to consider that, in the preparation of tissue and content samples, cell disruption might have resulted in the cross-contamination of samples, and the impact on the present results must be considered in future investigations.

According to assays using a cathepsin D substrate, a proteolytic activity is elevated in the tissue homogenates of the midgut (AMG and PMG), possibly due to this enzyme’s role in lysosomal digestion, but important levels are also shown in the PMG contents (Figure 2A). Furthermore, this activity had optimum pH values between 3 and 4 (Figure 2C), likewise with previous data obtained in *R. prolixus* (Terra et al., 1988), other Triatominae species (Balczun et al., 2012) as well as other hemipterans (Houseman and Downe, 1983b; Pimentel et al., 2017). However, in *Triatoma infestans*, the pH of the anterior and posterior midguts never decreased below pH 5.2 after blood ingestion (Balczun et al., 2012). The activity against this substrate is successfully abolished by pepstatin A, but complete inhibition is also shown by E-64, a highly selective cysteine protease inhibitor, and a partial inhibition by PMSF (Figure 2B). This mixed inhibition profile may have been caused by the use of crude samples, containing a complex mixture of active molecules and proteases, or even by the synergistic activity of different proteases against the substrate. The previous characterization of *R. prolixus* cathepsin D like digestive protease was carried out using hemoglobin as substrate (Terra et al., 1988), which could be hydrolyzed by various proteases, even in strongly acidic pH levels. Moreover, cathepsin D non-specific inhibition by E-64 has already been observed in other insect species (Padilha et al., 2009; Pimentel et al., 2017). In this case, the use of purified samples might reduce non-specific inhibitions.

Z-F-R-AMC is a well-known cathepsin L substrate (Terra and Ferreira, 2012). The majority of the activity against this substrate is located in *R. prolixus* PMG luminal fraction (Figure 1A), indicating a secreted protease activity in this compartment, which is suggested to be the true digestive midgut region (Garcia et al., 2007). The optimum pH at pH 5.0 (Figure 1C) is congruent with previous studies of *R. prolixus* cysteine protease using casein or N_α –_benzoyl-DL-arginine-β-naphthylamide hydrochloride (Terra et al., 1988). This activity was partially inhibited in the presence of pepstatin A (Figure 1B), a feature also observed in cathepsin from the spider *Nephilengys cruentata* (Fuzita et al., 2015). Pepstatin disrupts the digestion of *R. prolixus* nymphs fed with blood containing the compound, leading to reduction in molting and retarded oogenesis (Garcia et al., 1981), which might be due to its capability of inhibiting both major digestive proteases of this insect. Complete inhibition, however, is seen with the cysteine protease inhibitor E-64 and with PMSF, the serine protease inhibitor which can also reversibly inhibit cysteine proteases. Furthermore, cathepsin L is also characterized by activation by sulfhydryl agents, such as DTT (Terra and Ferreira, 2012), a property observed in *R. prolixus* posterior midgut cysteine protease (Figure 1B).

Another enzyme which may be involved in the hydrolyzation of Z-Phe-Arg-MCA in *R. prolixus* midgut is cathepsin B, which was already described in the gut of *T. infestans* (Kollien et al., 2004). Similarly to *R. prolixus* PMG, *T. infestans* PMG (therein named as “small intestine”) has an activity against Z-Phe-Arg-PNA that is induced after feeding, with maximal values at 5 and 10 daf. Besides that, this *T. infestans* activity is partially inhibited by E-64 and CA-074, and inhibition by CA-074, which is highly specific for cathepsin B, was associated to a protein of 33.2 kDa. Besides that, these authors have found one gene coding for cathepsin L and another for cathepsin B, and the expression of the last one was determined as gut-specific. Interestingly, the authors observed a constitutive pattern of expression that is similar to that observed for many *R. prolixus* cathepsin genes, showing that cathepsin B might also be involved in the digestion of triatomines.

In addition to endopeptidases, *R. prolixus* digestion of proteins also relies on exopeptidases (Terra et al., 1988). Previous investigations revealed the involvement of an aminopeptidase, associated with the microvilli, perimicrovillar membranes, and lysosomes of the wall of the posterior midgut (Billingsley and Downe, 1985). *R. prolixus* also possesses three different soluble aminopeptidase activities in the PMG luminal contents (Terra et al., 1988). Insect aminopeptidases are usually aminopeptidases N and are found as soluble enzymes in the luminal content of more basal insects, whereas in more apomorphic orders they are more often membrane-bound proteases in the microvilli of midgut cells (Terra and Ferreira, 2012). In this context, samples were also assayed against L-Leu-AMC, a fluorogenic leucine aminopeptidase substrate (Lee et al., 2010). The highest activity against this substrate is located in the AMG tissue (Figure 3A), which is congruent with the hypothesis of an intracellular or membrane-bound activity. *R. prolixus*, however, as a hemipteran species, would be more prone to have a high amount of soluble aminopeptidases (Terra and Ferreira, 2012).

The activity against L-Leu-AMC was maximal at pH 9 (Figure 3C), which was similar to other insect digestive aminopeptidases (7.2–9.0) and agrees with previously published data (Terra and Ferreira, 2012). This activity was equally reduced by PMSF and phenanthroline, while it was abolished by the combination of the two compounds (Figure 3B). In the coleopteran *Morimus funereus*, crude extract leucine aminopeptidase was entirely inhibited by pepstatin A and partially inhibited by both PMSF and phenanthroline, while for the purified enzyme the most efficient inhibitor was phenanthroline (Božić et al., 2008). Bestatin did not influence the *R. prolixus* activity (Figure 3B), in contrast to the high level of inhibition seen in other aminopeptidases (Lee et al., 2010). Amastatin, which is another leucine aminopeptidase inhibitor but also effective against aminopeptidase N, did not alter *R. prolixus* activity.

Even though all tested substrates showed some level of hydrolysis in all tested crude samples, the more significant proteolysis tends to be focused in the PMG luminal contents, except for the aminopeptidase activity, which showed higher levels in the AMG tissue. This data is somewhat congruent with the previous description of the PMG as the true digestive compartment of the gut of *R. prolixus*, which proposed the AMG as a portion in which erythrocyte lysis occurs due to a hemolytic factor (Garcia et al., 2007). Interestingly, each compartment and protease activity displayed a unique pattern of induction after the ingestion of blood.

### Cathepsin L-Like Activities

Intestinal cathepsin L activity is not influenced by abdominal stretching but induced by the concentration of proteins of the posterior midgut (Garcia and Garcia, 1977). Therefore, this protease has been described as an enzyme secreted into the lumen of the PMG of *R. prolixus* through a secretagogue mechanism related to the concentration of proteins in this compartment (Houseman et al., 1985). The protein levels in the AMG and PMG contents rapidly increased at 2 daf, accompanied by a decrease on the following days (Supplementary Figures 2E, 3A). However, this does not correlate to the proteolysis patterns measured for the cathepsin L activity in the same samples (Figures 4D,E). It is relevant to compare the change in the amount of proteins in the AMG from 2 to 14 daf (53 – 4.3 mg = 48.7 mg) to the total amount of protein in the PMG at 2 daf (∼0.5 mg), which suggests that, during this period, at least 97 cycles of filling and emptying the PMG occur (48.7/0.5). That would correspond to approximately eight cycles per day (97/12), meaning that the digestion of *R. prolixus* may follow short cycles of 3 h. This is an interesting possibility that may be approached in future studies.

Interestingly, in the PMG contents, cathepsin L possessed higher activities at 5 and 9 daf than that in unfed insects (Figure 4E). This observation, in addition to the observed expression patterns for C1 genes in the gut constitutive for most of the C1 genes preferentially expressed in the PMG, suggested that the control of these protease activities relies in a post-translational mechanism. Alternatively, the substrate used in the present study may not have revealed all the activities produced by the insect.

Regarding the expression of catepsin L genes coding for cathepsin L-like enzymes, four of the selected genes are differently expressed in the PMG (C1, C3, C4, and C10; Table 4). These genes showed higher levels of expression in the gut of adult females when compared to carcass samples and were also overexpressed in the PMG (Ribeiro et al., 2014). Therefore, these are probably the coding genes for the digestive cathepsin L-like in *R. prolixus.* Meanwhile, gene C8 is equally expressed in AMG and PMG samples, whereas transcriptomic sequencing found significantly more reads for this gene in PMG. Furthermore, genes represented by C6, C11, C14, and C15 were classified as non-digestive proteases, while the remaining genes were not found by previous authors (Ribeiro et al., 2014).

In search of the genes responsible for the profile of the proteolytic activity of cathepsin L against the fluorogenic substrate Z-F-R-AMC, we divided the digestive process into stages, starting with unfed male adults, followed by early (2–5 daf), intermediate (7–9 daf), and late (12–14 daf) digestion. The AMG overexpressed genes C1, C4, and C10 in an interesting temporal pattern of expression (Table 5). These three genes showed a significant drop in expression during the intermediate phase of digestion, while gene C3 showed a change in amplicon size depending on the feeding status of the insect (Table 5), which can be a result of an alternative splicing site triggered by blood ingestion (Supplementary Figure 11).

Interestingly, in the PMG, genes C3, C4, and C10 are constitutively expressed throughout the digestive process (Table 6), which suggests that these genes may not be involved in the changes in the proteolytic profile of PMG contents (Figures 7D, 8D, 9D). Meanwhile, gene C1 showed the same decline in PMG expression during intermediate digestion as seen in the AMG. Furthermore, there was no clear division regarding any of the lapses of time considered during blood digestion in the expression patterns of C1 genes. Thus, it remains elusive whether the digestive cathepsin L activity is generated by different expressions of the family C1 genes or whether, in the case of *R. prolixus*, this activity pattern might have resulted from translational or post-translational mechanisms. This is supported by the previous observation on the ultrastructural localization of *R. prolixus* midgut cathepsin L, with a strong correlation between the number of secretory vesicles originating from the Golgi apparatus and cathepsin L activity after feeding (Houseman et al., 1985).

### Cathepsin D-Like Activities

The highest cathepsin D activity levels were found in the PMG tissue and contents, which showed temporal patterns of activity similar to each other (Figures 5B,E) but differing in the cathepsin L pattern. This activity showed a single peak of activity at 5 daf, coinciding with higher activities of cathepsin L activity in PMG contents (Figure 4E). Therefore, it is clear that each type of protease may show a different expression profile depending on the tissue considered.

Notably, the AMG had previously been described as a compartment in which there was only absorption of water, erythrocyte hemolysis, hemoglobin crystallization, and hemozoin formation (Garcia et al., 2007; Ferreira et al., 2018). It has been hypothesized that the high levels of family A1 transcripts in the AMG might be caused by their synthesis as pro-enzymes, which would then be activated in the PMG (Ribeiro et al., 2014). However, we registered significant levels of enzyme activity in the luminal content of the AMG (Figure 5D). This activity may have escaped detection due to the lower sensibility of colorimetric assays using proteins as substrates. Some of the recorded transcripts may have intracellular roles in the AMG and especially in the HG, which is not characterized as a secretory organ (Ribeiro et al., 2014). This agrees with the high proteolytic activities observed in all tissue samples (Figures 5A–C).

In our study, most of A1 family genes also showed preferential expressions in the AMG (Table 2). In the AMG, several genes did not show any indications of changes in expression levels throughout digestion (A2, A3, A5, A8, A16, A17, and A18). However, genes A6, A10, A11, A14, and A15 probably have roles in intracellular processes, like autophagy, in the AMG of unfed insects, being more expressed in this period (Table 2). Nevertheless, A1, A4, A12, and A13 are probably involved in the initial hydrolysis of blood proteins since their expression levels increased in early digestion (Table 2). In the PMG, there was also a high number of genes constitutively expressed (Table 3). However, genes A5, A11, A12, and A13 were mainly expressed during early digestion in the PMG and could be responsible for the activity peak at 5 daf (Figures 5B,E).

### Aminopeptidases

The *R. prolixus* digestive aminopeptidase is highly associated with the gut wall, and its primary location would indicate a functional separation between this peptidase and the major digestive proteases (Terra et al., 1988). This functional separation would allow a higher environmental pH for maximal activity (Figure 3C), strongly differing from the more acidic pH range of the cathepsins. Besides that, final peptide hydrolysis would take place in the vicinity of the gut microvilli (Terra and Ferreira, 2012). *R. prolixus* aminopeptidase was cytochemically located in the microvilli surface, and it is associated to perimicrovillar membranes (Billingsley and Downe, 1985). In this respect, part of the activity measured in each tissue homogenate (Figures 6A–C) may be generated by membrane-bound or intracellular aminopeptidases since aminopeptidases have also been detected to be associated with the membranes of lysosomes, storage vesicles, and rough endoplasmic reticulum (Billingsley and Downe, 1985).

Tissue aminopeptidase activities were always higher when compared to their respective luminal contents, especially in the AMG (Figure 6). There is no aminopeptidase activity in any compartment before a blood ingestion, according to Billingsley and Downe (1985). However, according to our analyses, there is measurable activity in unfed insects (Figure 6), probably due to a higher sensitivity of the fluorescent assessment method. Earlier studies were unable to detect any activity associated with the AMG cells, leading to the erroneous conclusion that there are no digestive activities in this compartment (Billingsley and Downe, 1985), an information also dissonant with those presented herein. The gradual increase in aminopeptidase activity in the AMG tissue, which peaks at 7 daf, may be generated by the rise of membrane-bound enzymes, lysosomes, or even cellular metabolism after blood ingestion (Billingsley and Downe, 1985).

The activities of PMG aminopeptidases exhibit complimentary profiles when the tissue homogenates were compared to the lumen (Figures 6B,E). This observation suggests that secretory structures are involved in transporting aminopeptidase from lysosomes to the microvilli, as previously observed (Billingsley and Downe, 1985), increasing the amount of soluble enzymes. Furthermore, the luminal content exhibited a pattern consistent with previous descriptions of the activity in this compartment. The *R. prolixus* male adults aminopeptidase activity increased at a slower rate and reached maximal levels between 6 and 14 daf, according to Houseman and Downe (1983a). We observed an increase in activity between 5 and 9 daf (Figure 6E).

### Natural Substrates

For determination of the total proteolytic activity of the midgut of *R. prolixus*, samples were assayed with casein, which was the first substrate employed in the characterization of the digestive proteases of this insect, along with the two major blood proteins, namely, albumin and hemoglobin, in conditions which favored the activity of cathepsin L. Interestingly, each substrate resulted in a different pattern of activity during digestion. For instance, with hemoglobin, the activity after blood ingestion increased in a much slower rate in the luminal contents of the PMG and maintained steady levels during digestion (Figure 8D). Meanwhile, with bovine serum albumin, the activity rose abruptly and reached a peak at 5 daf (Figure 9D), coinciding with the increase in cathepsin L activity (Figure 4E). The levels then decreased gradually, differing from the cathepsin L fluorogenic data. Casein resulted in a different pattern in the PMG contents (Figure 7D), with peaks at 2 and 7 daf.

Taken together, our data suggest a possible division of protein digestion into temporal phases. The presence of a sequential temporal pattern would mean that alternate sets of cathepsin genes are being expressed at different time intervals during protein digestion. This pattern is noteworthy because the rate of protein decrease in the AMG seems to be constant throughout the digestion cycle (Supplementary Figure 3), which raises the hypothesis that the whole digestive process might be controlled by the passage of AMG contents to the PMG through the pyloric valve. However, the physiological mechanisms involved in the maintenance of this constant level of protein degradation through the expression of different sets of cathepsins are still unclear.

The different expression of gut proteases during blood digestion was already described in dipterans, such as the well-known early and late digestive trypsins of *Aedes aegypti* female adults (Felix et al., 1991). In *A. aegypti*, the transcription of early trypsin in females (Noriega et al., 1996) starts right after adult emergence from pupal stage, when the mRNA is also stored in the epithelium of the midgut. After blood ingestion, the translation of the early trypsin starts within the first hours. The second peak of protease activity is associated with the transcription and translation of late trypsin gene that is activated by the presence of a large number of proteins from the blood of the host, like albumin or gamma globulin (Noriega and Wells, 1999). This biphasic gene transcription and activity patterns have also been described in *Anopheles gambiae* (Dana et al., 2005) and *Culex quinquefasciatus* (Reid et al., 2015).

However, the striking differences between *R. prolixus* and mosquito digestion of protein-rich diets indicate independent adaptations for hematophagy. The transition from a predacious common ancestor of Heteroptera to hematophagy probably occurred in two distinct events within Hemiptera, once in Reduviidae, which includes the triatomines, and another in Cimicidae, which consists of bedbugs (Li et al., 2017). Although not much light has been shed previously to the proteolytic activity patterns of *R. prolixus*, stepwise cycles of activity have already been observed in fifth instar nymphs in the early stages of the digestive protease investigations, which corroborates our findings. The proteolytic activity against casein of these nymphs peaked at 6 daf (Garcia and Garcia, 1977). A second peak was then registered after 12 daf, which the authors connected to the occurrence of the ecdysis phase due to the overlapping of the two events. This delay of the two peaks, when compared to our data (Figure 7D), might be explained by the difference in duration of the digestive process of nymphs and adults. The same was seen by Houseman and Downe (1983a), however assessing the activity of cathepsin L against benzoyl-DL-arginine-*p*-naphthylamine instead of the more generalist substrate previously used. Furthermore, curiously, despite the coincidental timing of the second peak of proteolytic activity during the molting process of immature stages, the sequential pattern may be maintained even in adults, which reinforces the putative importance of this phenotype in nutrient acquisition. It is also relevant to consider that our assays of gut samples obtained along the digestive process did not include the activator DTT. This molecule restores the reduced form of the thiol catalytic group, and that may be important for the full assessment of activities in samples previously exposed to atmospheric oxygen (Terra et al., 1988). This methodological issue may account for differences observed between our data and previous reports. It would be important in the future to confirm the cathepsin L induction pattern observed here in activated samples, as the relative contribution between the activities in different time points observed might change.

### Final Remarks

The characterization of protease activity in *R. prolixus* revealed distinct tissue and temporal patterns. To our knowledge, this is the first description outside the order Diptera for different pools of proteases involved in initial, intermediate, or late stages of blood digestion. This may be an interesting case of evolutive adaptative convergence associated with independent adaptations for hematophagy that present different sequential patterns for protease expression in the insect gut. Contrasting patterns were clearly evidenced for the main digestive protease families in Triatomines: (i) cathepsin L-like and (ii) cathepsin D-like. This knowledge may allow the development of new control strategies for blocking the transmission of parasites harbored by kissing bugs, either by interfering directly with the gut physiology of the vector or by modulating the interactions between pathogens and digestive enzymes.

## Data Availability Statement

All datasets generated for this study are included in the article/Supplementary Material.

## Author Contributions

FG and BH conceived and designed the study. BH performed the experiments and data analysis. BG, PO, PA, and EG gave important intellectual contributions. All authors contributed to the article and approved the submitted version.

## Conflict of Interest

The authors declare that the research was conducted in the absence of any commercial or financial relationships that could be construed as a potential conflict of interest.
