# Supplementary material for: Characterization of the Temporal Pattern of Blood Protein Digestion in Rhodnius prolixus: First Description of Early and Late Gut Cathepsins
Source: Front Physiol. 2021 Jan 13;11:509310. doi: 10.3389/fphys.2020.509310 (PMC7838648; doi:10.3389/fphys.2020.509310)
Supplement: Supplementary file 1 [file Table_1.docx]

Supplementary Table 1. Final volumes of homogenates obtained from *R. prolixus* anterior midgut, posterior midgut, and hindgut. These volumes were used for more accurate calculations of the amounts of protein and enzyme activities per insect. daf– days after feeding. Controls without biological samples added to the saline solution resulted in measurements of 247 ± 1 and 500 ± 0 µL. Results were obtained from 5 (unfed) or 8 (other conditions) independent determinations.

| Sample | Unfed | 2 daf | 5 daf | 7 daf |
| --- | --- | --- | --- | --- |
| Anterior midgut contents | 487 ± 3 | 557 ± 5 | 525 ± 3 | 514 ± 1 |
| Anterior midgut tissues | 249 ± 2 | 249 ± 2 | 251 ± 2 | 250 ± 2 |
| Posterior midgut contents | 241 ± 3 | 245 ± 1 | 244 ± 1 | 246 ± 2 |
| Posterior midgut tissues | 256 ± 4 | 246 ± 2 | 250 ± 2 | 251 ± 1 |
| Hindgut contents | 246 ± 2 | 249 ± 2 | 246 ± 2 | 243 ± 1 |
| Hindgut tissues | 250 ± 1 | 249 ± 4 | 253 ± 1 | 254 ± 1 |
