# Supplementary material for: Characterization of the Temporal Pattern of Blood Protein Digestion in Rhodnius prolixus: First Description of Early and Late Gut Cathepsins
Source: Front Physiol. 2021 Jan 13;11:509310. doi: 10.3389/fphys.2020.509310 (PMC7838648; doi:10.3389/fphys.2020.509310)
Supplement: Supplementary file 2 [file Table_2.DOCX]

Supplementary Table 2. Vector Base Identification numbers of genes from protease Families A1 and C1 of *Rhodnius prolixus*. The table also shows the designed codes (this work), and number of cycles used for each gene for PCR amplification from samples of Anterior Midgut (AM), Posterior Midgut (PM) and Hindgut (HG).

|  |  |  | cycles | | |
| --- | --- | --- | --- | --- | --- |
| Family | Gene | Code | AM | PM | HG |
| A1 | RPRC002696 | A1 | 30 | 30 | 35 |
|  | RPRC004171 | A2 | 24 | 24 | 35 |
|  | RPRC010954 | A3 | 30 | 30 | 35 |
|  | RPRC012785 | A4 | 30 | 30 | 35 |
|  | RPRC006698 | A5 | 27 | 27 | 35 |
|  | RPRC011752 | A6 | 30 | 30 | 35 |
|  | RPRC012664 | A7 | 35 | 35 | 40 |
|  | RPRC012786 | A8 | 27 | 27 | 35 |
|  | RPRC015079 | A9 | 20 | 20 | 35 |
|  | RPRC00247_RPRC002479 | A10 | 20 | 20 | 35 |
|  | RPRC00433_SEQ-GL573083_RPRC010954 | A11 | 24 | 24 | 35 |
|  | RPRC00602_SEQ-GL562693_RPRC006290 | A12 | 20 | 20 | 30 |
|  | RPRC006759 | A13 | 30 | 30 | 35 |
|  | RPRC01474_SEQ-GL563145_RPRC014747_RPRC012504 | A14 | 20 | 20 | 24 |
|  | RPRC015076:RPRC015076 | A15 | 24 | 24 | 30 |
|  | RPRC015082 | A16 | 30 | 30 | 40 |
|  | RPRC008989 and RPRC012487 | A17 | 30 | 30 | 24 |
|  | RPRC01250_RPRC012513 | A18 | 30 | 30 | 35 |
| C1 | RPRC000294 | C1 | 30 | 20 | 24 |
|  | RPRC000405 | C2 | 30 | 30 | 35 |
|  | RPRC015289 | C3 | 35 | 20 | 24 |
|  | RPRC000205 | C4 | 30 | 20 | 24 |
|  | RPRC015299 | C5 | 35 | 30 | 40 |
|  | RPRC015288 | C6 | 35 | 30 | 40 |
|  | RPRC015290 | C7 | 30 | 30 | 35 |
|  | RPR006917-6907 | C8 | 24 | 20 | 30 |
|  | RPRC000309 | C9 | 35 | 30 | 35 |
|  | RPRC002593 and RPRC005321 | C10 | 30 | 20 | 24 |
|  | RPRC010398 | C11 | 30 | 30 | 30 |
|  | RPRC008250 | C12 | 30 | 30 | 40 |
|  | RPRC013528 | C13 | 30 | 27 | 30 |
|  | RPRC002640 | C14 | 27 | 27 | 30 |
|  | RPRC013182 | C15 | 27 | 24 | 35 |
|  | RPRC005322 | C16 | 35 | 35 | 35 |
