# Supplementary material for: Characterization of the Temporal Pattern of Blood Protein Digestion in Rhodnius prolixus: First Description of Early and Late Gut Cathepsins
Source: Front Physiol. 2021 Jan 13;11:509310. doi: 10.3389/fphys.2020.509310 (PMC7838648; doi:10.3389/fphys.2020.509310)
Supplement: Supplementary file 3 [file Table_3.DOCX]

Supplementary Table 3. Total amount of proteins in the contents of the anterior midgut of *Rhodnius prolixus* adult males at different times before and after blood feeding. daf – days after feeding.

|  | Protein total amount, mg |
| --- | --- |
| Unfed | 0.063 ± 0.007 |
| 2 daf | 53 ± 3 |
| 5 daf | 35 ± 4 |
| 7 daf | 26 ± 5 |
| 9 daf | 20 ± 2 |
| 12 daf | 6 ± 1 |
| 14 daf | 4.3 ± 0.9 |
