# Supplementary material for: Characterization of the Temporal Pattern of Blood Protein Digestion in Rhodnius prolixus: First Description of Early and Late Gut Cathepsins
Source: Front Physiol. 2021 Jan 13;11:509310. doi: 10.3389/fphys.2020.509310 (PMC7838648; doi:10.3389/fphys.2020.509310)
Supplement: Supplementary file 4 [file Data_Sheet_1.DOCX]

Supplementary Material


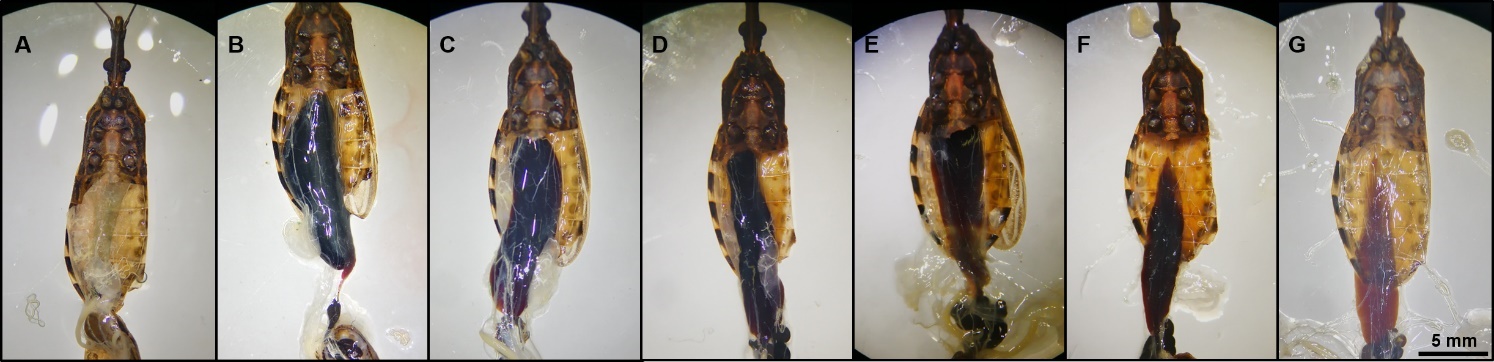


**Supplementary Figure 1.** Representative images of *Rhodnius prolixus* adult male digestive tract content pattern during blood digestion. A: fasting insect, B-G: 2, 5, 7, 9, 12 and 14 days after defibrinated rabbit blood intake, respectively (10x magnification).
