# Supplementary material for: Characterization of the Temporal Pattern of Blood Protein Digestion in Rhodnius prolixus: First Description of Early and Late Gut Cathepsins
Source: Front Physiol. 2021 Jan 13;11:509310. doi: 10.3389/fphys.2020.509310 (PMC7838648; doi:10.3389/fphys.2020.509310)
Supplement: Supplementary file 5 [file Data_Sheet_2.DOCX]

Supplementary Material





**Supplementary Figure 2.** Protein content (µg/µL) in the different digestive tract compartments of *R. prolixus* adult males while unfed and from 2 through 14 days after the ingestion of defibrinated rabbit blood. A: Anterior midgut (AMG). B: Posterior midgut (PMG). C: Hindgut (HG). D: Anterior midgut content (AMGc). E: Posterior midgut content (PMGc). F: Hindgut content (HGc). Figures are means ± SEM based on protein determinations carried out in 21 biological replicates obtained from pools of two insects each. In a dataset, groups with the same superscript letter are not significantly different (p>0.05). Consider different scalings of protein contents.
