# Supplementary material for: Characterization of the Temporal Pattern of Blood Protein Digestion in Rhodnius prolixus: First Description of Early and Late Gut Cathepsins
Source: Front Physiol. 2021 Jan 13;11:509310. doi: 10.3389/fphys.2020.509310 (PMC7838648; doi:10.3389/fphys.2020.509310)
Supplement: Supplementary file 6 [file Data_Sheet_3.DOCX]

Supplementary Material

**Supplementary Figure 3.** Protein concentration (µg/µL) and Rate of protein digestion (ug/uL.day) in the Anterior midgut contents of *R. prolixus* adult males from 2 through 14 days after the ingestion of defibrinated rabbit blood. A: Protein concentration. B: Rate of protein digestion. Figures are means ± SEM based on protein determinations carried out in 21 biological replicates obtained from pools of two insects each. In a dataset, groups with the same superscript letter are not significantly different (p>0.05).
