# Supplementary material for: Characterization of the Temporal Pattern of Blood Protein Digestion in Rhodnius prolixus: First Description of Early and Late Gut Cathepsins
Source: Front Physiol. 2021 Jan 13;11:509310. doi: 10.3389/fphys.2020.509310 (PMC7838648; doi:10.3389/fphys.2020.509310)
Supplement: Supplementary file 9 [file Data_Sheet_6.DOCX]

Supplementary Material





**Supplementary Figure 6.** Aminopeptidase specific activities (L-Leu-AMC as substrate) in the different digestive compartment tissues and luminal contents of *Rhodnius prolixus* male adults, unfed and at 2, 5, 7, 9, 12 and 14 days after feeding on defibrinated rabbit blood, assayed in buffer 0.2 M glycine-NaOH pH 9.0. A: Anterior midgut (AMG) tissues. B: Posterior midgut (PMG) tissues. C: Hindgut (HG) tissues. D: Anterior midgut contents (AMGc). E: Posterior midgut contents (PMGc). F: Hindgut contents (HGc). Figures are means ± SEM based on activity determinations carried out in nine different homogenate samples obtained from pools of two insects each. In a dataset, groups with the same superscript letter are not significantly different (p>0.05). Consider different scalings of activities.
