# Supplementary material for: Characterization of the Temporal Pattern of Blood Protein Digestion in Rhodnius prolixus: First Description of Early and Late Gut Cathepsins
Source: Front Physiol. 2021 Jan 13;11:509310. doi: 10.3389/fphys.2020.509310 (PMC7838648; doi:10.3389/fphys.2020.509310)
Supplement: Supplementary file 12 [file Data_Sheet_9.DOCX]

Supplementary Material





**Supplementary Figure 9.** The specific activities against bovine serum albumin (BSA) in the different digestive compartments tissues and luminal contents of *Rhodnius prolixus* male adults, unfed and at 2, 5, 7, 9, 12 and 14 days after feeding on defibrinated rabbit blood, assayed in buffer 0.2 M citrate-phosphate pH 5.5. A: Anterior midgut tissues (AMG). B: Posterior midgut tissues (PMG). C: Anterior midgut contents (AMGc). D: Posterior midgut contents (PMGc). Figures are means ± SEM based on activity determinations carried out in three different homogenate samples obtained from pools of two insects each. In a dataset, groups with the same superscript letter are not significantly different (p>0.05). Consider different scalings of activities.
