# Supplementary material for: Characterization of the Temporal Pattern of Blood Protein Digestion in Rhodnius prolixus: First Description of Early and Late Gut Cathepsins
Source: Front Physiol. 2021 Jan 13;11:509310. doi: 10.3389/fphys.2020.509310 (PMC7838648; doi:10.3389/fphys.2020.509310)
Supplement: Supplementary file 13 [file Data_Sheet_10.DOCX]

Supplementary Material


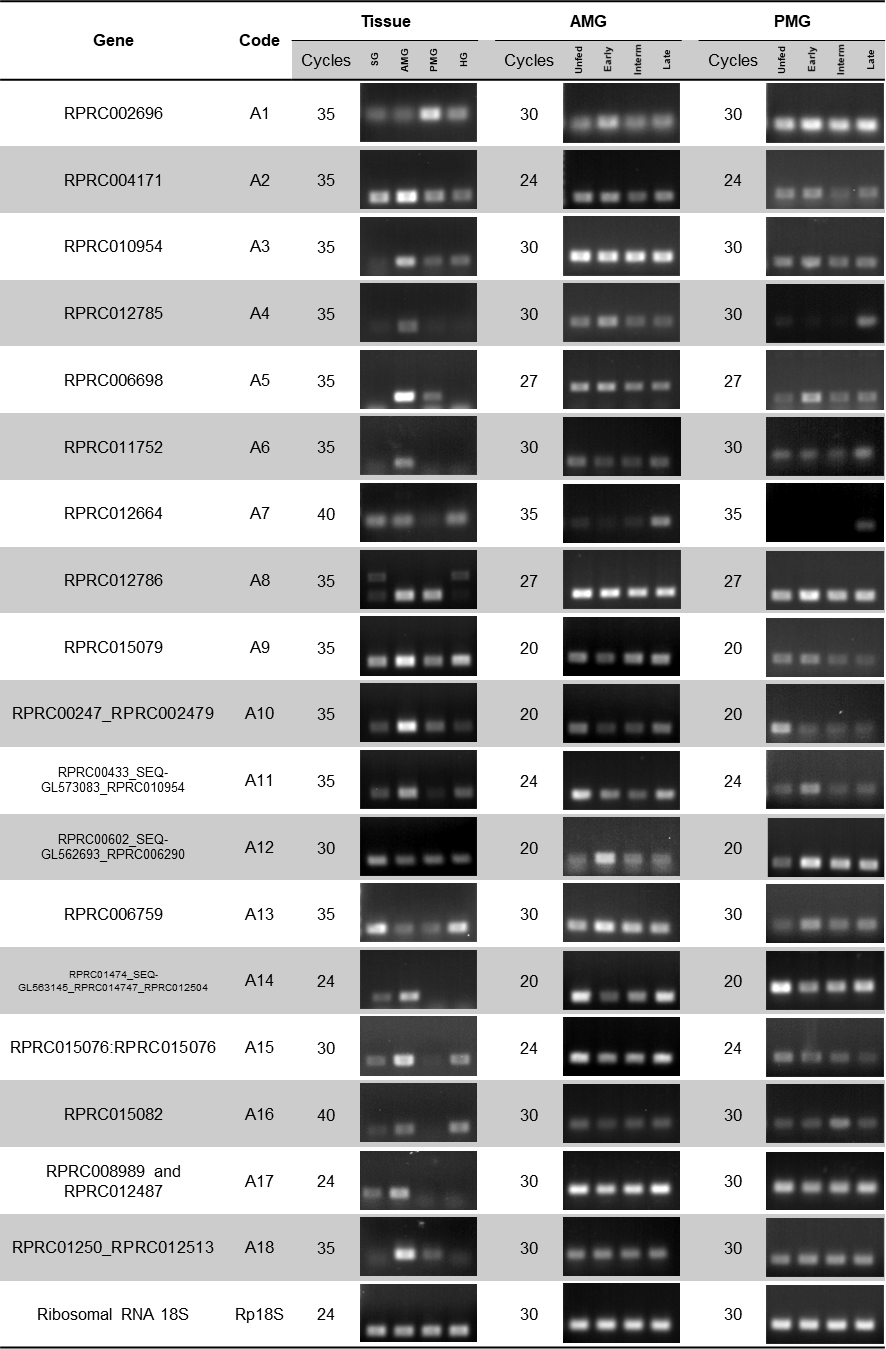


**Supplementary Figure 10.** Representative image of RT-PCR amplification products from *Rhodnius prolixus* male adults corresponding to selected protease family A1 (cathepsin D-like proteins) genes, including tissue and temporal expression patterns in the anterior midgut (AMG) and posterior midgut (PMG).
